# Microglia-Derived Brain Macrophages Associate with Glioblastoma Stem Cells: A Potential Mechanism for Tumor Progression Revealed by AI-Assisted Analysis

**DOI:** 10.3390/cells14060413

**Published:** 2025-03-11

**Authors:** Yuqi Zheng, Haneya Fuse, Islam Alzoubi, Manuel B. Graeber

**Affiliations:** 1 Ken Parker Brain Tumour Research Laboratories, Brain and Mind Centre, Faculty of Medicine and Health, University of Sydney, Camperdown, Sydney, NSW 2050, Australia; yzhe8012@gmail.com; 2 School of Medicine, Sydney Campus, University of Notre Dame, 160 Oxford Street, Darlinghurst, Sydney, NSW 2010, Australia; haneya.fuse@my.nd.edu.au; 3 School of Computer Science, The University of Sydney, J12/1 Cleveland St, Darlington, Sydney, NSW 2008, Australia; ialz9547@uni.sydney.edu.au; 4 University of Sydney Association of Professors (USAP), University of Sydney, Sydney, NSW 2006, Australia

**Keywords:** Iba1 (AIF1), brain macrophages, CD163, glioblastoma stem cells (GSCs), microglia, tumor microenvironment

## Abstract

**Background:** Malignant gliomas, and notably glioblastoma, are highly aggressive brain tumors. Understanding the mechanisms underlying their progression is crucial for developing more effective treatments. Recent studies have highlighted the role of microglia and brain macrophages in glioblastoma development, but the specific interactions between these immune cells and glioblastoma stem cells (GSCs) remain unclear. **Methods:** To address this question, we have utilized AI-assisted cell recognition to investigate the spatial relationship between GSCs expressing high levels of CD276 (B7-H3) and microglia- and bone marrow-derived brain macrophages, respectively. **Results:** Using PathoFusion, our previously developed open-source AI framework, we were able to map specific immunohistochemical phenotypes at the single-cell level within whole-slide images. This approach enabled us to selectively identify Iba1+ and CD163+ macrophages as well as CD276+ GSCs with high specificity and to study their co-localization. Our analysis suggests a closer association of Iba1+ macrophages with GSCs than between CD163+ macrophages and GSCs in glioblastoma. **Conclusions:** Our findings provide novel insights into the spatial context of tumor immunity in glioblastoma and point to microglia-GSC interactions as a potential mechanism for tumor progression, especially during diffuse tissue infiltration. These findings have significant implications for our understanding of glioblastoma biology, providing a foundation for a comprehensive analysis of microglia activation phenotypes during glioma development. This, in turn, may lead to new therapeutic strategies targeting the early stages of the immune microenvironment of glioblastoma.

## 1. Introduction

Microglia and brain macrophages are considered to play important roles in malignant glioma progression [1]. Microglial cells are resident to normal brain tissue and may turn into macrophages once fully activated, but brain macrophages can also derive from extraparenchymal sources, such as blood-borne myeloid cells, which is especially relevant in tumors [2]. Our recent study [3] has shown that microglia and microglia-derived brain macrophages are predominantly found in regions of glioblastoma infiltration, whereas macrophages of recent bone marrow origin are more prevalent in necrotic tumor areas. To elucidate the interactions between glioblastoma stem cells (GSCs) and the tumor microenvironment, we applied Artificial Intelligence (AI)-assisted cell recognition to whole-slide images. Our goal was to determine whether GSCs exhibiting high CD276 (B7-H3) expression preferentially co-localize with microglia-derived brain macrophages or with recently infiltrated blood-derived myeloid cells.

Findings obtained from diseased human tissues hold great relevance in medical research, as they directly reflect the complex biology of human diseases. However, certain studies are not feasible using fixed and archival clinical specimens, highlighting the need for complementary approaches. Experimental animal studies have proven particularly helpful. Normal resident microglia are highly ramified cells and easily recognizable in immunohistochemical stains such as Ionized Calcium-Binding Adapter Molecule 1 (Iba1). This also holds true in many central nervous system (CNS) diseases, but a problem arises once microglia have become so activated that they undergo transformation into full-blown phagocytes that closely resemble macrophages invading from the bloodstream. Experimental animal models have provided us with invaluable information about microglial phenotypic and functional plasticity under those conditions. The examination of tissue dynamics has revealed that not all local microglia transform simultaneously when challenged by tissue pathology [4]. Thus, a gradient of microglial activation is typically visible in brain tissue affected by disease processes. This explains why microglia-derived macrophages may retain some residual branching of cell processes, as discussed later in the text. The vast majority of Iba1 immunoreactive macrophages in brain tissue can be shown to be derived from microglial cells under such controlled conditions, whereas CD163 and its animal homologues, e.g., ED2 in rats, do not appear to label most microglial cells.

Iba1, the Ionized Calcium-Binding Adapter Molecule 1, also known as Allograft Inflammatory Factor 1 (AIF1), is a widely recognized microglia/macrophage marker that belongs to the EF-hand protein superfamily [5]. Iba1 plays a critical role in calcium-mediated signaling pathways, including those involving Ras-Related C3 Botulinum Toxin Substrate (Rac) GTPase and Phospholipase C (PLC)-γ [6,7]. The downstream effects of these pathways include cytoskeletal reorganization, phagocytosis, cellular activation, and inflammatory responses [8,9], with Iba1 expression being upregulated in response to IFN-γ stimulation [10,11]. Increased levels of Iba1 have been found to correlate with GBM tumorigenesis and a reduction in patient survival time [12]. The Iba1 antibody not only works on paraffin-embedded material but also across several species, enabling highly informative comparative studies.

CD163 (also known as M130), a member of the Scavenger Receptor Cysteine-rich (SRCR) superfamily, is specifically expressed by monocytes and macrophages, and its levels are more frequently elevated in GBM compared to healthy brain tissue. CD163+ cells may exhaust T cell responses via Interleukin (IL)-10 released downstream of the Janus Kinase (JAK)/Signal Transducer and Activator of Transcription (STAT) pathway, which may contribute to the immunosuppressive nature of GBM [13]. CD163 expression on monocytes and macrophages can be modulated by various cytokines. Proinflammatory cytokines such as Tumor Necrosis Factor (TNF)-α, Interferon (IFN)-γ, and lipopolysaccharides (LPS) attenuate the expression of CD163, while anti-inflammatory cytokines like IL-6 and IL-10 upregulate it [14,15]. CD163 is a well-established marker for identifying monocyte-derived macrophages, including in tumors, where they form part of the population of tumor-associated macrophages (TAMs) [16,17,18,19]. CD163-positive macrophages are considered to contribute to tumor progression, especially by promoting angiogenesis, tumor cell proliferation, and survival.

In glioblastoma (GBM), a subset of the cancer cell population exhibits neural stem cell (NSC)-like characteristics, along with tumor-initiating capabilities and resistance to therapies [20]. These glioblastoma-initiating cells (GICs), also referred to as GSCs, play a crucial role in GBM progression and are closely related to NSCs [21]. These cells appear to express high levels of CD276 [22]. CD276, also known as B7-H3, is a co-stimulatory molecule with immunoinhibitory effects on T cell activation and proliferation [23,24]. Its expression is typically low in normal tissues but elevated in various cancers [25,26]**,** and CD276 overexpression has been linked to adverse patient outcomes in several cancer types [25,27], including glioblastoma [28,29]. Previous studies have shown that CD276 upregulation promotes the expression of stem cell markers, such as Prominin 1 (PROM1/CD133) [30], CD44, and POU Class 5 Homeobox 1 (POU5F1/Oct4), potentially facilitating the epithelial–mesenchymal transition (EMT) in certain cancers [31,32].

PathoFusion, our previously developed AI framework [22,33,34], permits the recognition of pathomorphological features and the mapping of immunohistochemical data after training a bifocal convolutional neural network (BCNN). This method has been extended to allow the detection and analysis of arbitrarily defined single cells within whole-slide images [22,33,34]. Using this powerful new technology, we have specifically studied the tissue distribution of Iba1+ and CD163-expressing macrophages in human glioblastoma biopsies in relation to CD276+ GSCs, with the aim of gaining insights into their in situ relationship.

## 2. Materials and Methods

### 2.1. Wet Laboratory Work

**Figure 1:**
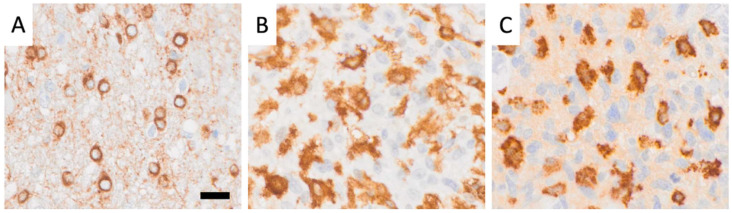
Representative images of (**A**) CD276-expressing GSCs, (**B**) Iba1-immunoreactive resident microglia, characterized by rounded macrophage-like morphology and short, stout processes, and (**C**) CD163-positive blood-derived macrophages, distinguished also by their limited ramification. The PathoFusion framework was trained to selectively identify all three cell types. Scale bar: 20 μm.

Whole slide images (WSIs) of a cohort of 33 human glioblastoma cases were used. The clinical characteristics of these cases have been published [3] and are shown in Table 1. In brief, paraffin sections were immunohistochemically labeled for Iba1 (AIF1), CD163, and CD276 (B7-H3), as described [3], and scanned using an Olympus VS-120 scanner (VS120 Virtual Slide System, Olympus, Japan). Only the resulting WSIs were used for this study. Representative images of CD276-expressing GSCs, Iba1-immunoreactive resident microglia-derived macrophages with short, stout cell processes, and CD163-positive macrophages are shown in Figure 1. Iba1-positive macrophages, which typically still show at least some ramifications, are assumed to be derived from resident microglia, whereas the majority of CD163-positive cells are considered to be recently blood-derived based on what is known about the expression of these markers and extensive work also in animals. The PathoFusion framework, which is further explained in the following, was trained to accurately identify all three cell types.

### 2.2. The PathoFusion Framework

**Figure 2:**
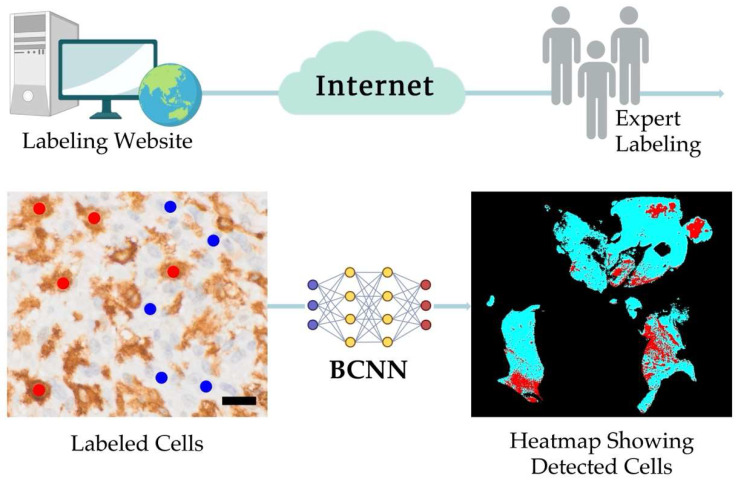
Schematic illustration of the PathoFusion framework featuring a web-based online labeling platform that enables researchers to annotate cell populations. Cells of interest are marked with red dots (as shown for a selection of Iba1-immunoreactive microglia/macrophages), while non-relevant cells or tissue elements are annotated with blue dots for exclusion. The bifocal neural network (BCNN) then generates whole-slide heatmaps for each marker, predicting the distribution of immunoreactive cells (CD276, Iba1, CD163) of the morphological subtype selected within the respective tissue section. These heatmaps must not be confused with the fusion maps in this study. Scale bar: 20 μm.

Our PathoFusion framework [22,33,34] was utilized to analyze immunohistochemically stained WSIs. This framework employs a two-step approach: cell detection and segmentation. In brief, it employs a deep learning model (BCNN) to identify cells within the WSIs applying trained criteria. The framework then uses threshold segmentation and edge detection techniques to isolate individual cells from their surrounding tissue. This permits the automated identification of defined morphological subsets of cells even in complex staining environments [22]. For the present study, the network was specifically trained to recognize Iba1 and CD163 labeled macrophages in addition to CD276 immunoreactive GSCs. The CD276 dataset has been reported previously [22]. A schematic illustration of the PathoFusion framework is shown in Figure 2.

### 2.3. Dataset Preparation, Partitioning, Filtering, and Thresholding for CD276, Iba1, and CD163 Immunoreactive Cell Analysis

**Figure 3:**
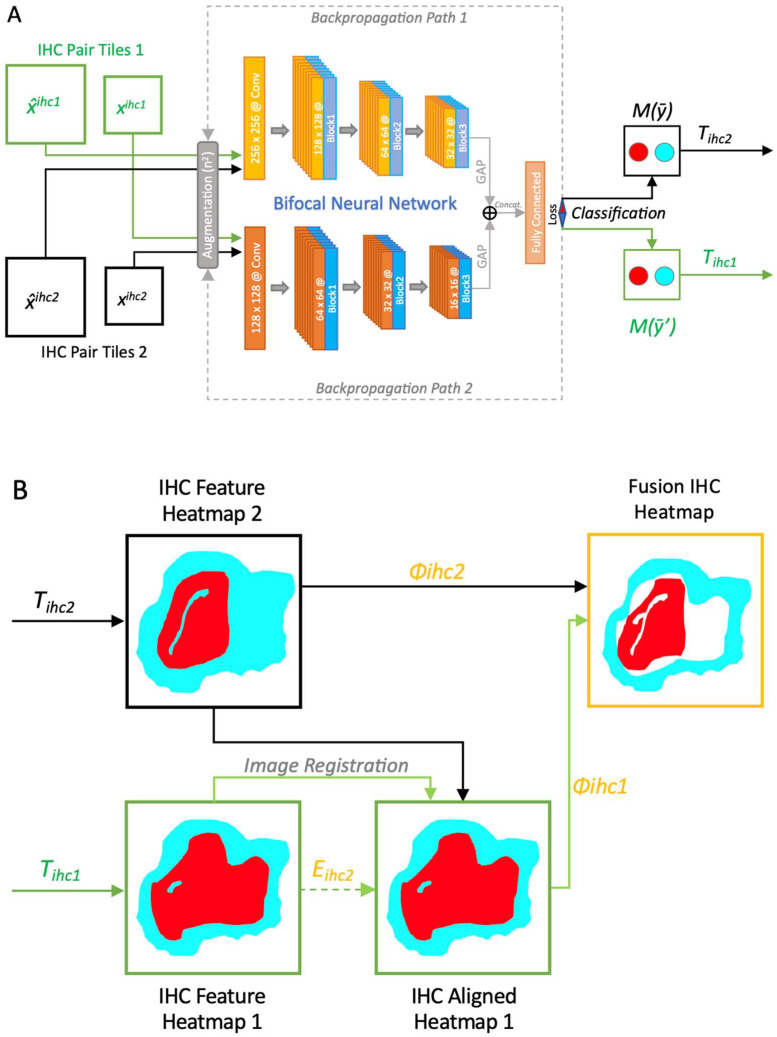
(**A**) Schematic representation of how the Bifocal Convolutional Neural Network (BCNN) is trained for cell recognition using paired immunohistochemistry (IHC) image patches. The BCNN consists of two processing pathways that enable the network to capture detailed features from localized cellular regions while also considering broader contextual information. (**B**) Schematic representation of how the Bifocal Convolutional Neural Network (BCNN) is trained for cell recognition using paired IHC image patches, contd. The output feature heatmaps highlight the regions of an image that contribute most significantly to the model’s classification decisions; regions marked in red are of interest, while turquoise indicates regions where all cells of interest carrying the respective marker are absent in both images. The resulting fused heatmaps display the overlapping areas of CD276 expression with CD163 and Iba1 in red, indicating spatial correlation, while white areas signify the absence of such correlation.

We extracted 32 × 32 and 64 × 64-pixel image patches from annotated coordinates on 32 immunochemically labeled whole-slide images, each divided into four non-overlapping tiles (Figure 3). The BCNN model was trained on small image fragments (patches) featuring each type of cell individually (Figure 3A), including CD276-positive GSCs (Figure 1A), round, macrophage-like Iba1-positive microglial cells (Figure 1B), and CD163-immunoreactive macrophages (Figure 1C). These patches containing Iba1+ microglia/macrophages and CD163+ macrophages were marked by “red dots”, indicating the presence of cells of interest, while cells or features without immunolabeling were marked by “blue dots” (Figure 2). These patches were split into training/cross-validation and testing sets: CD276 (36,734 paired patches for training, 14,005 for testing), Iba1 (20,064 paired patches for training, 5016 for testing), and CD163 (15,186 paired patches for training, 3796 for testing), ensuring exposure to heterogeneous tissue variations and staining artifacts. For evaluation, patches were extracted from whole-slide images with a 10-pixel stride. To improve cell discriminability, we employed a multi-step image processing approach. This approach enhances the visibility and distinctness of target cells (Iba1, CD163, and CD276) through image enhancement, contrast enhancement, and automatic thresholding. Our bifocal design incorporated multi-inputs from 32 × 32-pixel patches for cell-specific features and 64 × 64-pixel patches for broader tissue context, enabling comprehensive feature extraction and improved cell discrimination. This approach successfully mitigated errors such as boundary ambiguities and overlapping regions. We plan to further optimize the model with adaptive feature extraction strategies to address challenges in detecting faintly stained cells.

The following computational work, which is illustrated in Figure 3, was performed: The BCNN model was trained on high resolution sample images taken from the WSIs, featuring CD276-positive GSCs, round, macrophage-like Iba1-positive cells, and CD163-immunoreactive macrophages. The BCNN consists of two processing pathways that enable the network to capture detailed features from small areas, focusing on individual cells, while also taking contextual information in the surroundings of these cells into account [34]. To enhance detection precision and model robustness, we employed a multi-step image processing approach, including color normalization [35], background subtraction, and edge enhancement filters to address staining variability and improve cell boundary detection. Image augmentation techniques such as rotation, contrast, and sharpness adjustments were applied to the training dataset, which enhance the diversity of the training dataset and improve generalization. This increased variability allows the BCNN to process a broader range of training samples and reduces the likelihood of overfitting. Global Average Pooling (GAP) is employed to extract features from processed IHC patches, condensing these features of special interest into a more compact form for subsequent classification. The model merges features extracted from different layers, capturing both cellular-level details and contextual information simultaneously via concatenation (*concat.*; Figure 3A). The discrepancy between the predicted class probabilities generated by the BCNN and the actual labels of the IHC images is characterized by the loss function, quantifying the error of misclassification by the model. The classification is provided by the output of *M(ȳ)*, which assesses the IHC staining intensity for each cell type, allowing for objective classification based on their staining characteristics (Figure 3A). The output layer of the BCNN model classifies cells into categories defined by a threshold function (*T*) (Figure 3), based on their IHC staining profiles. 

**Figure 4:**
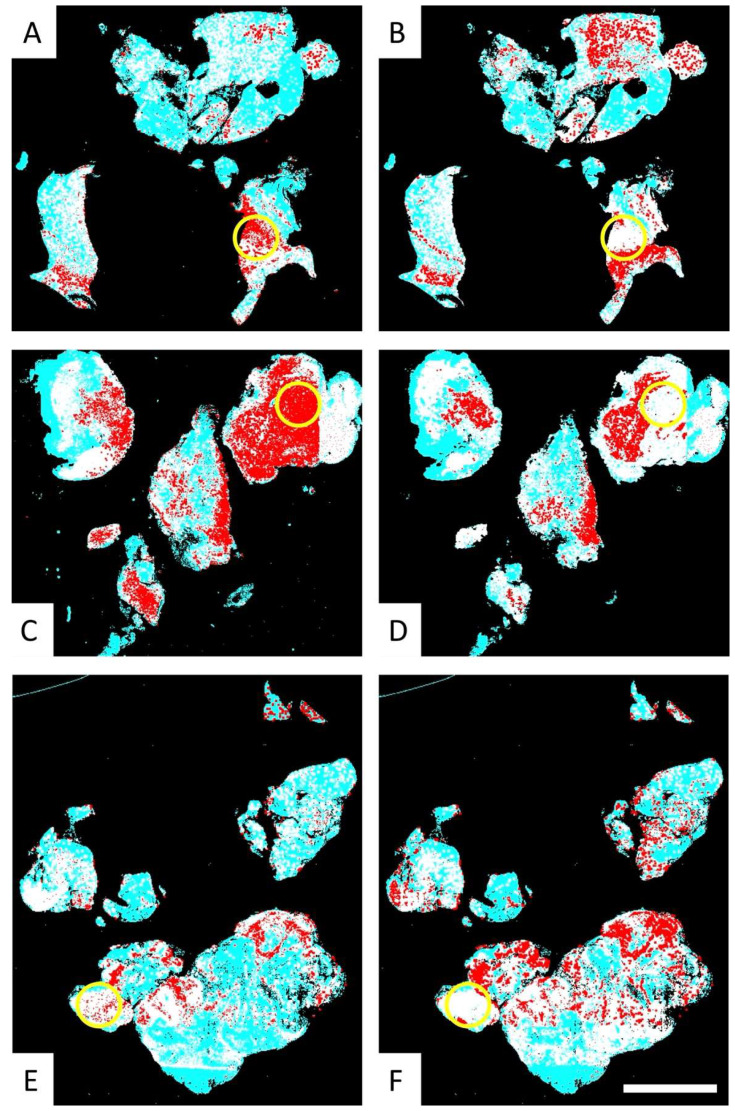
Fusion heatmaps of CD276 with Iba1 (**A**,**C**,**E**) and CD163 (**B**,**D**,**F**) illustrate the co-localization of CD276-positive GSCs with microglia/macrophages expressing these markers. It is essential to recognize that all immunolabeling shown reflects the presence of cells detected with AI-assistance, i.e., cells that exhibit the morphology defined for training the network. In other words, the underlying pre-fusion heatmaps are selective for the predefined cell types and therefore differ from conventional immunohistochemical heatmaps. Areas of co-localization are represented in red on the fusion heatmaps, whereas tissue regions with discrepant localizations are shown in white. For turquoise see Figure 3. Yellow circles highlight examples of biopsy areas where Iba1-immunoreactive macrophages, but not CD163-expressing macrophages, associate with GSCs. Scale bar: 5 mm.

The output feature heatmaps highlight the regions of an image that contribute most significantly to the model’s classification decisions; regions marked in red indicate higher importance, while turquoise indicates less significance. The alignment of the output feature maps generated by the BCNN with corresponding spatial locations in the heatmaps, which represent regions enriched for cell types in the IHC WSIs, is achieved through image registration using the distance matrix *E* (Figure 3B). The fusion heatmaps are generated by combining feature heatmaps into a single, unified output using the function *Φ*. Finally, the fusion heatmaps allow for the simultaneous visualization of CD276 expression and Iba1 and CD163 labeling, respectively, in a single combined whole-slide image for each case. Thus, the resulting fused heatmaps display the overlapping areas of CD276 expression with Iba1 and CD163 in red, indicating spatial correlation, while white areas represent regions where one image shows cells of interest present while the other does not, and turquoise areas represent regions where all cells of interest carrying the respective marker are absent in both images (Figure 3B and Figure 4). It is important to note that all representations refer to the detected cell populations of interest, but not all immunohistochemical staining results. This critical focus is owed to AI assistance and would not be possible using conventional microscopic methods.

### 2.4. Post-Processing Steps

Following cell identification and thresholding by the BCNN model (Figure 3), we applied post-processing steps to additionally quantify key cellular features. These steps included edge and contour detection techniques for further segmentation and precise measurements of CD276+, Iba1+, and CD163+ cells. The following steps were applied: edge detection identified areas with significant pixel intensity changes, typically at cell borders; contour detection identified closed curves connecting pixels of identical intensity, outlining cell shapes by tracing their borders; thresholding converted cell images to binary format (white: cells; black: background), enhancing contour detection by making cell borders more distinct. We also quantified cell features by calculating mean values, including cell area, perimeter, compactness, and minimum pixel intensity.

## 3. Results

### 3.1. Morphological Tissue Analysis Is Greatly Aided by AI Assistance

Immunohistochemistry has been around for several decades, and a number of widely used cellular markers exist, such as Iba1 and CD163, for CNS microglia and myeloid macrophages, respectively (Figure 1B,C). However, systematic morphological fine analysis of selected phenotypic subtypes within a population of immunolabeled cells in situ requires additional methodology. We have used an AI-assisted approach in this study to selectively focus on cells with a rounded macrophage morphology. This was possible following the training of a bifocal CNN to recognize these cells (Figure 3). The same AI model was successfully used to selectively analyze strongly CD276 immunoreactive GSCs in a highly complex staining environment because CD276, unlike Iba1 and CD163, is not a cell-type specific marker. Examples of representative images of CD276-expressing GSCs (Figure 1A), as previously described by Alzoubi et al. [22], Iba1-immunoreactive microglia/macrophages, and CD163-positive macrophages used for training of PathoFusion are shown in Figure 1.

### 3.2. The PathoFusion Framework Allows Visualization of the Locations of GSCs and Microglia/Macrophages on the Basis of Multimodal Heatmaps

Following training, the PathoFusion framework pinpointed the locations of the different cell types, allowing a unique view of the presence of highly activated microglia with macrophage morphology and infiltrated myeloid cells in relation to each other and to the location of GSCs. We are not aware of any other technology allowing the co-correlation of arbitrarily defined morphological subtypes of cells. This allowed us to visualize the detected cells of interest by means of heatmaps (AI-detected cells are visualized in red on a turquoise background; Figure 2), showing the relative distribution of CD276, round Iba1, and CD163-positive cells throughout entire slide scans. The fusion heatmaps revealed the precise tissue distribution and co-localization of CD276-expressing GSCs with round Iba1-positive microglial cells, as well as CD163-positive macrophages (Figure 4). Given the limited size of the cohort following selection (Table 1), our approach focused on qualitative assessments and visual evaluation of Iba1+ and CD163+ macrophage distributions and their spatial correlation with GSC using heatmaps. These visualizations provided valuable insights into tissue heterogeneity and immune cell distribution patterns. Future work will incorporate comprehensive quantification and statistical analysis to further validate and strengthen these observations based on the use of a much larger cohort.

### 3.3. Fusion Heatmaps Reveal Regional Differences Between the Localization of CD276+ GSCs and Iba1+ and CD163+ Macrophages

Fusion heatmaps allow for the simultaneous visualization of CD276 expressing cells of interest with Iba1 and CD163 labeling in a single WSI per case (Figure 3B). The resulting fused heatmaps display the overlapping areas of cellular expression for CD276, Iba1, and CD163 in red, indicating spatial correlation, while white areas signify the absence of such correlation (Figure 4). Interestingly, we observed regional differences between the localization of CD276+ GSCs with round/amoeboid Iba1+ microglia/macrophages and CD163+ macrophages, respectively. There was a striking absence of CD163-positive macrophages in areas where activated Iba1-expressing microglia co-localize with CD276-positive GSCs (Figure 4; examples are indicated by yellow circles). This highlights significant intra-tumoral heterogeneity of Iba1 and CD163 expression among glioblastoma biopsies, in keeping with a recent study on glioblastoma necroses [3]. Our PathoFusion model selectively detects morphological macrophage-like Iba1- and CD163-positive cells while disregarding ramified cells according to its training for the current study.

### 3.4. Comparative Analysis of the Multimodal Heatmaps Indicates That Microglia-Derived Brain Macrophages Are More Frequently Associated with Glioblastoma Stem Cells than Myeloid Cells

**Figure 5:**
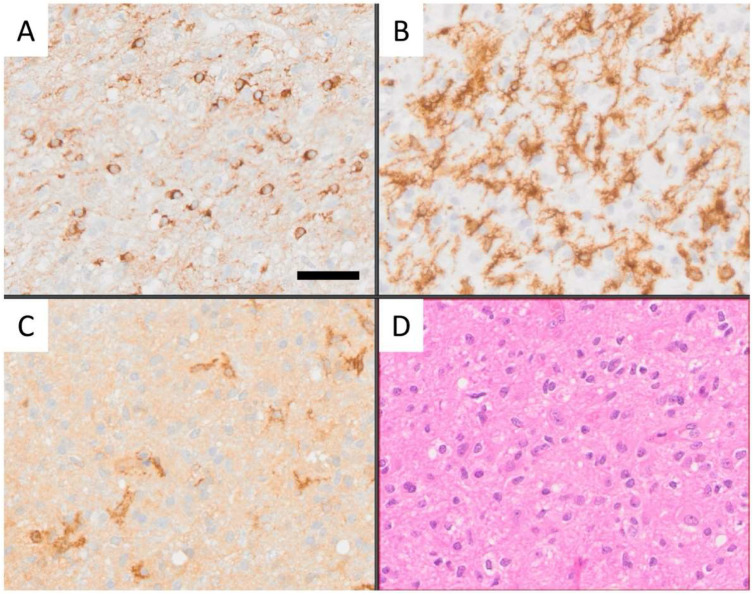
Conventional histological images of the immunostains used are shown for comparison, focusing on the tissue areas marked by the yellow circles in Figure 4C,D, respectively. These images show an example of co-localization of CD276-positive GSCs with Iba1-immunoreactive microglia in a region of glioblastoma with diffuse tumor cell infiltration (**D**). CD276-positive GSCs, (**A**); Iba1-positive reactive microglia and macrophages; a number of microglia still displaying ramified morphology, (**B**); only a few CD163-positive cell processes are present in this region, and none are round, explaining the negative fusion signal, (**C**); routine Hematoxylin and Eosin (H&E) staining showing diffusely infiltrating glioma, (**D**) Scale bar: 50 μm.

Figure 5B,C illustrate the differences in the localization of reactive Iba1+ microglia/macrophages compared to CD163+ macrophages, explaining the differences in expression of Iba1 and CD163 observed in the selected heatmap area. Our results indicate that Iba1+ microglia/macrophages are more frequently located in proximity to CD276-expressing GSCs (Figure 5A) than CD163 expressing myeloid cells. Notably, in areas devoid of CD163 macrophages, processes/fragments of CD163+ cells can be observed (Figure 5C).

## 4. Discussion

The primary objective of this study was to answer the question of whether there exists a spatial relationship between infiltrating GSCs, as identified by strong cytoplasmic CD276 expression [22], and the presence of microglia-derived macrophages and/or macrophages of myeloid origin. In order to answer this question, we used our recently developed AI framework, PathoFusion [22,33,34]. This new method allowed us to selectively identify highly activated Iba1-expressing microglia, i.e., cells expressing a morphological macrophage phenotype, and CD163-positive macrophages, which are predominantly of myeloid origin.

Our study reveals a preferential association between microglia-derived macrophages and GSCs, compared to myeloid macrophages. This finding is intriguing, as it suggests that interactions between microglia-derived macrophages and GSCs may play a role in glioblastoma progression. The spatial relationship between these cell types in situ has been understudied so far, despite the growing evidence of “crosstalk” between them. The significant tissue heterogeneity of glioblastoma biopsies presents a challenge for sample selection and analysis. To address this, we applied stringent neuropathological criteria, resulting in 15 case triplets out of the original 33 cases selected, where adjacent tissue sections labeled for all three markers were available and suitable for comparative AI-assisted analysis. Our primary goal was not to conduct a detailed examination of all microglial morphological phenotypes in glioblastoma, which is a future objective, nor to perform detailed measurements of the spatial associations between microglia/macrophages and myeloid macrophages with GSCs, respectively. Such an endeavor would require a much larger case series, allowing for extensive stratification of the cohort to account for the substantial heterogeneity of glioblastoma biopsies. Therefore, our study should be considered a proof-of-concept investigation rather than a quantitative analysis. However, our findings suggest that most, if not all, GBM biopsies contain CD276-high-expressing GSCs, although the numbers appear to vary significantly between cases depending on tissue composition. Importantly, these GSCs tend to associate with microglia-derived rather than myeloid macrophages.

### 4.1. Molecular Similarities Between GSCs and NSCs

**Figure 6:**
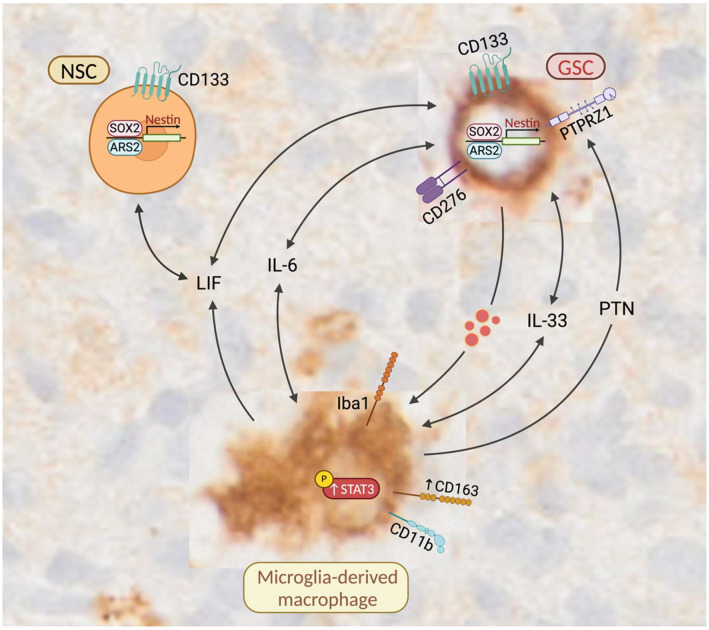
Proposed bidirectional “crosstalk” between GSCs and Iba1-immunoreactive microglia/macrophages. Molecular mimicry between NSCs and GSCs is a possibility. Both GSC and NSC populations can form neurospheres and express key stem cell markers, including CD133 (PROM1), ARS2 (SRRT), SOX2, and Nestin. Microglia-derived leukemia inhibitory factor (LIF) significantly upregulates Nestin expression in NSCs. IL-33 produced by glioma cells and a subset of Iba1+ microglia enhance phosphorylated STAT3 (p-STAT3), which may lead to increased expression of downstream factors such as LIF and IL-6 in both cell types. Elevated IL-6 levels can induce an anti-inflammatory phenotype in TAMs by upregulating CD163, which seems rare but principally also possible in microglia. Iba1 and CD163-positive glioma-associated microglia/macrophages produce large amounts of pleiotrophin (PTN). The receptor for PTN, protein tyrosine phosphatase receptor type Z1 (PTPRZ1), is predominantly expressed by GSCs. *Red dots:* GSC-derived exosomes containing p-STAT3. The tissue background was selected for illustration purposes. “Created with BioRender.”

NSCs are primarily found in the neurogenic niches of the adult brain and have been shown to regulate critical stem cell properties that support their self-renewal. Studies suggest that NSCs in the subventricular zone (SVZ) that have acquired initial oncogenic alterations may be the cells of origin for GBM [36,37,38,39]. There are molecular cell surface similarities between GSCs and NSCs, which may help GSCs invade brain tissue. For instance, both cell populations are capable of forming neurospheres and express key stem cell markers like CD133 (PROM1), Nestin, and SRY-Box Transcription Factor 2 (SOX2) [20,21,40,41,42,43]. It is now evident that Serrate RNA EffectorMolecule Homolog (SRRT), also known as Arsenic Resistance Protein 2 (ARS2), functions as a key transcription factor in NSC self-renewal, maintaining the multipotent progenitor state by directly activating the pluripotency gene SOX2 [44,45]. A recent study by Yin et al. has demonstrated that ARS2 regulates the stem cell-like properties of GSCs, including Nestin expression and sphere-forming ability [46]. ARS2 in GSCs mediates Monoglyceride Lipase (MGLL)/Prostaglandin E2 (PGE2)/Catenin Beta 1 (β-Catenin) downstream signaling, further promoting an immunosuppressive phenotype in macrophages via upregulation of CD163 and Arginase 1 (ARG-1). Vinel et al. investigated the similarities between GSCs and NSCs. Their study demonstrated that patient-matched induced NSCs (iNSCs) derived from expanded potential stem cells (EPSCs) exhibit comparable methylome and transcriptome profiles to glioblastoma-initiating cells (GICs). The authors showed that iNSCs can be utilized to predict drug responses, potentially enabling the identification of patient-specific therapeutic targets [47]. This approach offers a promising avenue for developing personalized treatment strategies in glioblastoma. Some molecular similarities between GSCs and NSCs are highlighted in the schematic illustration of Figure 6.

### 4.2. Co-Localization of Microglia-Derived Brain Macrophages and NSCs Is a Normal Feature of the SVZ

A number of studies suggest that glioblastoma may originate from NSCs, e.g., in the brain’s SVZ [37,38,39]. Furthermore, Wang et al. demonstrated that co-culturing GBM-derived extracellular vesicles (EVs) with NSCs led to the de-differentiation of NSCs into highly proliferative, migratory, tumor-promoting cells, in contrast to naïve NSCs [48]. Digregorio et al. have also demonstrated that a subset of GBM cells that express CD276 is capable of migrating from the primary tumor and invading the SVZ [49]. This finding aligns with the results of an earlier study, which identified a subpopulation of GBM cells expressing Nestin and Sox2 that not only demonstrated tropism towards the SVZ but are more tumorigenic than GBM cells within the tumor mass [20]. By using multiplex immunofluorescence (mIF), a technique which allows the detection and quantification of both cellular expression and co-localization of proteins in a tissue specimen [50], Wang et al. have shown that the expression of CD276 and Iba1 are correlated in glioma (*p* < 0.001, R^2^ = 0.13) [51]. Our PathoFusion heatmaps (Figure 4A,C,E) show examples of diffuse glioma growth where strongly Iba1+ immunoreactive microglia-derived macrophages are found in spatial proximity to CD276+ GSCs, outnumbering instances where CD163 and CD276+ cells co-occur. This co-localization may suggest active communication between these cell types, perhaps similar to the microglia neighboring NSCs in the SVZ, where such crosstalk has been shown to occur physiologically [52,53,54].

### 4.3. Microglia That Reside in Close Proximity to NSCs in the SVZ Are Characterized by a More Amoeboid Morphology

Microglial cells are a component of the neurogenic niche in the SVZ where they are often found in close proximity to NSCs [21,55]. Microglia residing in proximity of NSCs in a neurogenic microenvironment (SVZ) exhibit a unique phenotypic profile characterized by a more amoeboid morphology with enlarged cell somata with fewer, thickened cell processes and low expression of common microglia markers such as Iba1 and CD68 [56,57]. In contrast, aging microglia of the SVZ undergo significant phenotypic changes [58] and exhibit increased expression of the microglial markers, Iba1 and CD68, as well as enhanced secretion of pro-inflammatory cytokines, including IL-6, IL-1β, and TNFα [52,53]. These findings suggest that upregulation of Iba1 in microglia may directly influence the crosstalk with other cells. However, we found that Iba1 is significantly upregulated in GBM biopsies (Figure 1B and Figure 5B), and CD276+ GSCs appear to be more frequently accompanied by Iba1-expressing reactive microglia (Figure 5A,B) than CD163 immunoreactive macrophages, as indicated by our data. This may suggest that CD276+ GSCs modulate nearby resident microglia.

### 4.4. GSCs May Enhance the Expression of CD163 in Tumor-Associated Macrophages

De Boeck et al. have shown that IL-33 derived from glioma cells and a subset of Iba1+ microglia increases phosphorylated STAT3 (p-STAT3), potentially upregulating the expression of downstream LIF and IL-6 in both cell types [59,60]. It is worth noting that resident microglia and NSC-derived LIF is crucial for long-term NSC maintenance in the neurogenic niches such as SVZ [61,62]. Notably, IL-33 plays a significant role in the activation and recruitment of microglia, highlighting the bidirectional communication between glioma cells and Iba1+ microglia/macrophages (Figure 6). Importantly, elevated levels of IL-6 in the GBM microenvironment appear to have dual effects, i.e., inducing an anti-inflammatory phenotype in macrophages by upregulating CD163 [63,64] and sustaining stem cell-like properties in GSCs [65,66]. Similar to IL-33 and its downstream mediator IL-6, CD276 may have a role in the activation of STAT3 downstream signaling, which enhances stem-like characteristics in glioma cells and induces an anti-inflammatory phenotype in TAMs [67,68]. Importantly, CD276 expressed by glioma cells/GSCs may influence the differentiation of tumor-associated macrophages, i.e., increase the production of IL-10 and Mannose Receptor C-Type 1 (MRC1/CD206) while inhibiting Major Histocompatibility Complex, Class II, DR Alpha (HLA-DRA) expression on macrophages [69]. Gabrusiewicz et al. have demonstrated that exosomes derived from GSCs, which primarily contain p-STAT3, can induce CD163 expression in Integrin Subunit Alpha M (ITGAM/CD11b)+ monocytes [70]. Intriguingly, CD11b is a marker readily expressed by microglia, it was in fact the first known microglia activation marker [71], and CD11b+ microglia are capable of taking up GSC and NSC-derived exosomes [52,70] (Figure 6). As shown in Figure 5B, Iba1+ microglia-derived macrophages located in proximity to CD276-positive GSCs appear highly activated, and some cells even show incipient CD163 expression. GSCs exhibit a remarkable ability to hijack developmental pathways and manipulate the surrounding microenvironment, which is a key aspect of their malignant behavior. By exploiting signaling molecules such as ARS2, IL-6, LIF, and STAT3, GSCs may influence nearby microglia and create a supportive niche that may promotes their own maintenance and survival (Figure 6).

### 4.5. CD163-Expressing Macrophages Promote the Self-Renewal and Maintenance of GSCs

Shi et al. have found that CD163-positive glioma-associated macrophages produce large amounts of pleiotrophin (PTN) [72]. The protein tyrosine phosphatase receptor type Z1 (PTPRZ1), which serves as the receptor for PTN, appears to be predominantly expressed by GSCs [72]. PTN was found to increase GSC counts in cell viability tests and enhanced tumor sphere formation. These findings suggest that PTN may promote self-renewal and maintenance of GSCs through PTN–PTPRZ1 paracrine signaling. Furthermore, the interaction between PTN and PTPRZ1 triggers phosphorylation of downstream tyrosine substrates, which may initiate signaling cascades that promote cell survival, enhance adhesion, and stimulate migration [73]. Shi and colleagues have also observed that glioma tissue areas with extensive Iba1+ TAM infiltration showed increased PTN staining [72], suggesting that both Iba1+ microglia and CD163+ microglia-derived macrophages may play a role in supporting the survival and migration of GSCs; this paracrine signaling is tentatively visualized in Figure 6.

Notably, PTN enrichment has been observed in the SVZ, where NSCs exhibit tumor-homing properties and likely serve as the primary source of PTN. In vivo studies employing shRNA-induced PTN knockdown have revealed a significant decrease in glioma infiltration towards the SVZ [74]. Interestingly, Bhaduri et al. have identified an “outer radial glia-like” subset of GSCs, which express PTPRZ1 and contribute to the invasive behavior of glioblastoma [75]. Outer radial glial cells are found to be abundantly distributed within the outer region of the SVZ in the developing human brain [76]. Taken together, these studies point to potential bidirectional communication between CD276+ GSCs, Iba1+ microglia, and CD163-expressing macrophages, which may promote GSC invasion.

## 5. Conclusions

Resident microglia are more likely to play a role in the diffuse growth of GBM, as their association with GSCs was not found near necrotic tumor areas. Targeting the ability of GSCs to hijack developmental pathways and manipulate their surrounding microenvironment may be a promising therapeutic strategy for improving treatment outcomes. In addition, disrupting the interaction between GSCs and microglia could prevent the creation of an immunosuppressive niche that favors GSC maintenance and survival. Targeting the highly activated macrophage-like microglia in glioblastoma directly or modulating their phenotype may provide an effective approach to weakening the potential symbiotic relationship between GSCs and microglia, especially during phases of diffuse tissue infiltration. Thus, the results of our study emphasize the need for more research into the mechanisms underlying GSC-microglia interactions. A large series of glioblastoma cases, stratified for clinical parameters, should be used to carry out a quantitative assessment of our qualitative findings considering the structural complexity of glioblastoma tissue.

## Figures and Tables

**Table 1:** Clinical characteristics of glioblastoma cases.

Case	Sex	Age at Diagnosis	Survival (Months)	Localization
1 *	F	62	16	Right occipital
2 *	M	55	14	Left occipital, parietal
3 *	F	46	10	Left frontal
4 *	M	70	4	Right frontal
5	F	54	95	Left parietal
6	M	33	15	Right frontal
7 *	F	57	4	Right frontotemporal
8	F	48	27	Right frontal, occipital, parietal, temporal
9 *	M	65	14	Right frontal
10 *	M	69	16	Right frontal
11	M	51	23	Left parietal
12 *	F	55	12	Left frontal
13	F	85	4	Left temporal
14	F	72	13	Right temporal
15 *	M	77	20	Right parietal
16 ^1^	M	33	15	Right frontal
17	F	51	Unknown	Right frontal
18	M	50	16	Left temporal
19	F	60	20	Right temporal
20	F	75	27	Right frontal
21 *	M	65	10	Left temporal
22	M	33	8	Right frontal
23	F	60	21	Left temporal
24	M	68	10	Left frontal
25	F	59	38	Right occipital
26 *	F	79	12	Right parietal
27	M	73	17	Right frontal
28 *	M	50	20	Right parietal
29 *	M	66	12	Right parietal
30 *	M	50	15	Right temporal
31	M	78	5	Right frontal
32	F	60	15	Right occipital
33 *	M	75	16	Right occipital
34	F	62	13	Right occipital, parietal

Reference: [3]. Note: ^1^ Same as Case 6 (subsequent biopsy); * cases fulfilling all criteria for inclusion in the heatmaps.

## Data Availability

The datasets generated and/or analyzed during the current study are available from the corresponding author on reasonable request.
